# Health-Promoting Behaviors among Older Adults with Noncommunicable Diseases in Rural and Urban Areas during the New Normal Post-COVID-19 Era: A Structural Equation Modeling Analysis

**DOI:** 10.3390/nu15010101

**Published:** 2022-12-25

**Authors:** Wanich Suksatan, Supat Teravecharoenchai, Jintana Sarayuthpitak

**Affiliations:** 1 Department of Public Health, Faculty of Liberal Arts, Krirk University, Bangkok 10220, Thailand; 2 Department of Curriculum and Instruction, Faculty of Education, Chulalongkorn University, Bangkok 10330, Thailand

**Keywords:** COVID-19 pandemic, health-promoting behaviors, older adults, nutrition, NCDs

## Abstract

This study aimed to develop and test a causal relationship among perceived self-efficacy (PSE), health literacy (HL), access to COVID-19 preventive material (ACPM), social networks (SN), and health-promoting behaviors (HPBs). Multistage stratified random sampling was used to recruit 250 older adults with noncommunicable diseases (NCDs) from Thai urban and rural communities. The data were collected with self-reported questionnaires. Data analyses used descriptive statistics and structural equation modeling. The results indicated that participants in urban communities had higher PSE, ACPM, HL, SN, and HPBs than rural participants. The fitness parameters of the modified model (χ^2^ = 71.936, *df* = 58, *p*-value = 0.103, χ^2^/*df* = 1.240; root mean square error of approximation (RMSEA) = 0.031; standardized root mean square residual (SRMR) = 0.042; goodness of fit index (GFI) = 0.964; normed-fit index (NFI) = 0.964; comparative fit index (CFI) = 0.993) indicated its suitability as the research model. HPBs were directly positively influenced by PSE (β = 0.40, *p* < 0.001), ACPM (β = 0.24, *p* < 0.001), HL (β = 0.19, *p* < 0.01), and SN (β = 0.01, *p* < 0.05). Therefore, taking all predicting variables together could explain 81.0% of the variance in HPBs. Multidisciplinary healthcare teams could use these findings to establish proper interventions or healthcare activities to increase HPBs among older adults, particularly in this era of the “new normal”.

## 1. Introduction

The coronavirus disease 2019 (COVID-19) began a pandemic at the end of December 2019 and has caused severe damage to cities around the world [1]. Vaccination with the accelerated COVID-19 vaccine has been mandated worldwide to prevent the spread of COVID-19 [2,3]. However, the development and spread of the Delta and Omicron variants of COVID-19 could reduce the effectiveness of the vaccine and jeopardize efforts to contain the pandemic [4,5]. After approximately 3 years, 642 million people have had confirmed cases of COVID-19, and there have been 6.62 million deaths across the globe. In Thailand, there has been a cumulative total of 4.7 million people with confirmed cases and 33,285 deaths due to COVID-19 [6]. Since it began, the COVID-19 pandemic has had an impact on all people and in diverse areas, including the economy, society, and health-related well-being [7].

Numerous studies have found that older adults were disproportionately affected by COVID-19 during the global Omicron wave [8]. Older adult populations were regarded as being at greater risk of developing serious illness from COVID-19 than were younger people [9]. Prior risk analyses also revealed that older adults with noncommunicable diseases (NCDs), such as chronic kidney disease, diabetes, cancer, and heart conditions, might be more susceptible to developing severe illness [9]. However, several countries, including Thailand, have attempted to prevent and control the spread of COVID-19 by promoting healthy lifestyle behaviors, such as hand washing, mask wearing, social distancing, and avoiding crowded places [10]. In the “new normal” of the post-COVID-19 period, the Thai government has helped people realize that healthy lifestyles are the key to resilience in the fight against COVID-19 and numerous other health threats [10]. Health promotion aims to improve the health of all generations at all times, rather than just focusing on emergencies; however, COVID-19 may aid in driving the message home [11].

Health-promoting behaviors (HPBs) are particularly important for older adults with NCDs because they are effective methods of maintaining appropriate self-care. They also actively promote health-related well-being and better quality of life [12]. Pender’s health promotion model is regarded as an essential tool for determining health status, preventing disease, and enhancing health and well-being throughout a person’s lifetime [13], especially for epidemic control measures (e.g., social distancing, self-isolating, or home-sheltering) that disrupt the delivery of various types of health services. A global survey by the World Health Organization revealed that health treatments and prevention services for people with NCDs were significantly reduced or discontinued due to the COVID-19 pandemic [14]. A sustained and prolonged epidemic may affect HPBs, thereby diminishing the efficacy of COVID-19 controls and making it more difficult to maintain the lifestyles and well-being of older adults with NCDs [15,16]. These factors related to recent events have led to older adults becoming more aware of individual and public health. Improving HPBs by maintaining good health through stress management and a diet of foods that boost immunity can help protect this population from contracting this and other viruses.

Although several studies assessed HPBs during the first and second waves of the COVID-19 outbreak, most previous studies focused on the HPBs of hospitalized patients [17] and community-dwelling older adults [12,18]. Empirically, prior studies reported that factors associated with HPBs among older adults include perceived self-efficacy (PSE) [12,19], health literacy (HL) [20,21], access to COVID-19 preventive material (ACPM) [20], and social networks (SN) [18]. Consequently, these elements need to be accounted for when considering HPBs among this population. To the best of our knowledge, no study has captured any causal relationships or conducted a comparative study of urban and rural communities of older adults with NCDs with regard to HPBs in the new normal post-COVID-19 period. Consequently, this gap must be filled through the discernment of a causal relationship of HPBs that addresses these specific variables in older adults with NCDs. We selected the factors that can be modified by healthcare providers, including PSE, HL, ACPM, and SN, as the influencing factors of HPBs. With those selections, this study aimed to compare these variables between rural and urban older adults with NCDs as well as develop and test a hypothesized causal model of HPBs among older adults with NCDs who lived in rural and urban Thai communities post-COVID-19. In this new normal, it is necessary to have a better understanding of these factors and how they affect HPBs and other people, especially when there are differences, in order to guide and establish interventions that improve health and well-being among older adults in different residential areas.

## 2. Materials and Methods

### 2.1. Research Design

**Figure 1:**
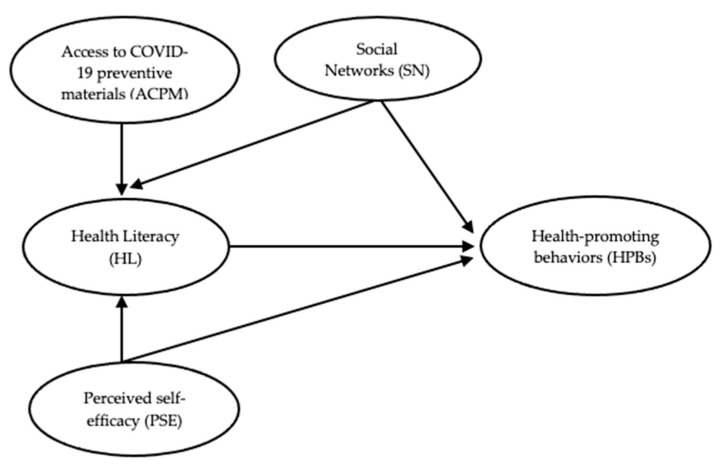
Research framework for the study.

The cross-sectional research design allowed this study to determine the consistency of the causal relationship model for PSE, HL, ACPM, SN, and HPBs using empirical data and examine the effects of these factors on HPBs among older adults with NCDs (see Figure 1). The data from “Determinants of the Health-Promoting Behaviors among Community-Dwelling Older Adults with Non-Communicable Diseases during the New Normal Post-COVID-19 Era” was used to obtain the aims of the present study.

### 2.2. Setting and Participants

Thailand defines an older adult as a person aged 60 years or older [22], and the urban and rural communities are classified according to residential areas [23]. The participants in this study were older adults with NCDs from Ubon Ratchathani city municipality (urban communities) and Huaruea and Nong Khon sub-districts (rural communities) in Ubon Ratchathani province in northeast Thailand and were recruited for this study via multistage stratified and simple random sampling selections. The study sample comprised (1) Thai older adults aged 60 years or more who had been (2) diagnosed with one or more NCD (i.e., vascular disease, heart disease, chronic obstructive pulmonary disease, diabetes, hypertension, cancer, and obesity) and (3) who could understand and communicate in Thai. Participants who were unable to complete the questionnaire due to visual/sensory/auditory abnormalities or who were unwilling to participate in this study were excluded. The number of possible sample sizes needed for a study with the structural equation model ranges from 100 to 800 [24]. We determined a suitable sample size using G*Power software version 3.1.9 [25] and determined that the effect size was 0.15 [26], with an alpha level of 0.05 and a power of 0.80. We added 30% to counterbalance any incomplete questionnaires and missing data, resulting in a final sample size of 250.

### 2.3. Research Instruments

The following six research instruments were used to collect the data:

Sociodemographic Data Form. This form, developed by the researchers, included four multiple choice or open-ended questions asking about the participant’s age, sex, monthly income, and type of NCD.

Health Literacy Scale (HLS). This measure was originally developed in Thai by the Health Education Division [27]. It comprises 10 items, and each item is answered on a 4-point ratings scale, with scores ranging from 1 (strongly disagree) to 5 (strongly agree). The total score is 10–50, and mean scores are divided into four levels: bad (10–19), fair (20–29), good (30–39), and very good (40–50), with higher scores indicating higher levels of literacy on health [27]. The psychometric properties of the HLS were tested and found to be valid and reliable [27]; the Cronbach’s alpha coefficient of this scale was 0.90 for this study.

Lubben Social Network Scale (LSNS-6). This measure was originally developed by Lubben et al. [28] and comprises six items with two subscales: family and friendships. Each item is answered on a 6-point rating scale with scores ranging from 0 (none) to 5 (9 or more). The total score is 0–30, and mean scores are divided into two levels: social isolation (0–12) and social engagement (13–30), with higher scores indicating more social engagement [28]. After the construct validity of the LSNS-6 was tested using confirmatory factor analysis (CFA), six items with two subscales remained and fit with the empirical data [28]. The Cronbach’s alpha coefficient of the LSNS-6 in this study was 0.88.

Self-Rated Abilities Scale for Health Practice (SRAHP). This measure was originally developed by Becker et al. [29]. It comprises 28 items with 4 subscales: nutrition, stress management, exercise, and health practice. Each item is answered on a 5-point ratings scale, with scores ranging from 0 (not at all) to 4 (completely). The total score is 0–112, and mean scores are divided into three levels: low (0–37), fair (38–74), and high (75–112), with higher scores indicating a higher level of self-efficacy [29]. After the construct validity of SRAHP was tested using CFA, four factors remained and fit with the empirical data [29]. The Cronbach’s alpha coefficient of SRAHP in this study was 0.95.

Access to COVID-19 Preventive Materials (ACPMS). This measure was originally developed in Thai by Yodmai et al. [20]. It comprises 5 items, with each item answered on a 3-point rating scale. Scores range from 0 (no or not sure) to 1 (yes), with a total score range of 0–5. The mean scores are divided into two levels: bad (0–2) and good (3–5), with higher scores indicating greater access to COVID-19 preventive materials [20]. Psychometric properties of ACPMS were tested and found valid and reliable [20]. The Cronbach’s alpha coefficient of ACPMS in this study was 0.76.

Health-Promoting Behaviors Scale (HPBS). This measure was originally developed by the Thai Health Education Division [27]. It comprises 19 items with 7 subscales: nutrition, exercise, smoking, alcohol drinking, stress management, rational drug use, and preventing COVID-19 infection. Each item is answered on a 5-point rating scale, with scores ranging from with 1 (not at all) to 5 (completely). The scores of HPBs are divided into four levels: bad (<60%), fair (60–69%), good (70–80%), and very good (>80%), with higher scores represent more HPBs. The Cronbach’s alpha coefficient of HPBS in this study was 0.75.

### 2.4. Data Collection

After submitting the required permission to participate and informed consent, eligible participants were able to access the self-reported questionnaire between 10 September and 10 November 2022 during the “new normal” post-COVID-19 era. Participants took approximately 30–40 min to answer all of the questions, and a total of 250 participants completed the questionnaire (100%).

### 2.5. Data Analyses

Statistical Package for Social Sciences (SPSS) version 25.0 and AMOS (analysis of moment structure) software were used for data analyses. The demographic data and all variables were assessed using descriptive statistics. Pearson’s correlation and Spearman’s rank correlation coefficient were used to examine the relationships between the measured variables. The independent t-test was used to compare the differences between rural and urban older adults with NCDs with regard to PSE, HL, ACPM, SN, and HPBs. Additionally, a structural equation model (SEM) was used to determine the causal relationships. Prior to the data analysis, all relevant assumptions were met. The fit of the hypothesized model was assessed based on several criteria, including (a) chi-square test (χ^2^, *p* > 0.05); (b) normed chi-square (χ^2^/*df*) with the desired value of <3; (c) the value of the root mean square error of approximation (RMSEA), which was ≤0.05; (d) the value of the comparative fit index (CFI), which was ≥0.90; (e) the normed-fit index (NFI), which was ≥0.90; (f) the value of standardized root mean square residual (SRMR), which was ≤0.08; and (g) the goodness-of-fit index (GFI), which was ≥0.90 [30]. The significance level was set at *p* < 0.05 for all analyses.

## 3. Results

### 3.1. Characteristics of Participants

The demographic data for the 250 participants revealed the following: 63.60% were female, 52.80% were aged 60–69 years (mean = 69.61, SD ± 7.47), 82.80% were generally uniformly spread on monthly income of less than USD 143 (mean = USD 132.14, SD ± 239.20), and 65.60% participants were living with hypertension. In addition, participants’ ages, education levels, and monthly income were positively significant with HPBs (*p* < 0.05), as shown in Table 1.

### 3.2. Perceived Self-Efficacy, Health Literacy, Access to COVID-19 Preventive Material, Social Networks, and Health-Promoting Behaviors among Urban and Rural Older Adults with NCDs

In this study, the participants had high levels of PSE (mean = 76.48, SD ± 17.55), and the SN indicated higher social engagement levels (mean = 15.08, SD ± 5.59). HL (mean = 37.28, SD ± 6.37), ACPM (mean = 37.28, SD ± 6.37), and HPBs (mean = 67.44, SD ± 7.51) were also all at good levels. In comparisons of the mean of PSE, HL, ACPM, SN, and HPBs, it was found that PSE, HL, ACPM, SN, and HPBs were significantly higher among participants who lived in urban communities than those in rural ones. We also found that all domains of PSE and HPBs were significantly higher for urban dwellers than rural respondents (see Table 2).

### 3.3. Structural Model

An SEM was used to evaluate the causal relationships based on the constructed framework and the null hypothesis. The results revealed that the obtained fit indices were χ^2^ = 556.163, *df* = 85, *p*-value = 0.000, χ^2^/*df* = 6.543; RMSEA = 0.149; SRMR = 0.166; GFI = 0.773; NFI = 0.719; CF = 0.749. We found the causal relationship model indicated that some statistical criteria were at unacceptable levels based on the empirical data [30]. Therefore, the model fit needed to be modified by adjusting the errors of several observed variables to allow relationships between them to increase the fit index values to an acceptable level.

**Figure 2:**
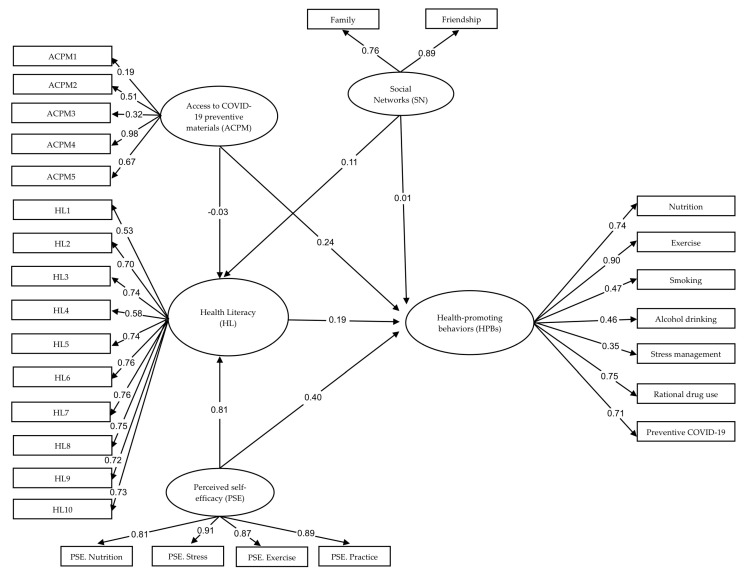
Causal relationship model of HPBs among the older adults with NCDs in our study.

After the adjustment, the model fit of HPB indices were acceptable, with the empirical data of χ^2^ = 71.936, *df* = 58, *p*-value = 0.103, χ^2^/*df* = 1.240; RMSEA = 0.031; SRMR = 0.042; GFI = 0.964; NFI = 0.964; and CFI = 0.993. The model explained 81.0% of the total variance in HPBs among the older adults with NCDs in our study (see Table 3 and Figure 2).

The standardized coefficients of the final model of HPBs showed that HPBs among older adults with NCDs was directly influenced positively by PSE (β = 0.40, *p* < 0.001), ACPM (β = 0.24, *p* < 0.001), HL (β = 0.19, *p* < 0.01), and SN (β = 0.01, *p* < 0.05). Therefore, all of the predicting variables were kept in the model and, all together, could explain 81.0% of the variance in HPBs.

Regarding indirect causal effects on HPBs, the results of the final model revealed that the three predictors of PSE (β = 0.15, *p* < 0.01), SN (β = 0.02, *p* < 0.05), and ACPM (β = −0.01, *p* < 0.05) indirectly affected HPBs via HL. The finding showed that HL was a mediator of HPBs among older adults with NCDs. In addition, the three variables altogether explained 72.0% of the variance in HL.

## 4. Discussion

To the best of our knowledge, this is the first study to directly examine the associations between the levels of PSE, HL, ACPM, SN, and HPBs and compare those variables in older adults with NCDs. This study also aimed to develop and test a hypothesized causal model of HPBs among older adults with NCDs who lived in Thai rural and urban communities during the “new normal” post-COVID-19 era.

In this study, we also determined the causal relationship, and our findings revealed that PSE is the most influential factor affecting HPBs among older adults with NCDs. This is because PSE increases an individual’s self-confidence and self-care skills; thus, people with higher PSE can improve their HPBs [13]. The results of this study are consistent with prior studies reporting PSE influences on HPBs among older adults in Thailand [12], Korea [21], Indonesia [31], and the United States [32]. PSE is a significant factor in improving motivation and, thereby, engagement in HPBs among older adults with NCDs. This means that individuals with higher PSE scores are better able to motivate themselves to engage regularly in HPBs [33]. A recent systematic review of self-efficacy and self-care among people with hypertension revealed that there were 21 studies across the globe, including in Africa, Asia, Australia, Europe, the Middle East, and the United States, reporting that higher PSE was related to self-care behaviors, such as physical activity, dietary changes, and medication adherence [34]. The findings also revealed significantly higher PSE scores in urban communities than in rural areas. The current findings are in line with previous studies that older adults who have a high level of PSE could improve their ability to perform HPBs. In fact, it has been found that older Thai adults with hypertension who live in urban areas have higher PSE and HPB scores than their peers in rural areas [12].

Additionally, we found that HL was directly affected by HPBs among older adults with NCDs. These findings support Do and colleagues’ multi-institutional study that examined HL and health behaviors among older adults during the COVID-19 pandemic [35]. They found that HL was associated with health-related behaviors, which included 8% more healthy eating behaviors (95% CI: 1.04–1.13), 4% more physical activity (95% CI: 1.01–1.08), and 9% less depression (95% CI: 0.87–0.94) [35]. From Nutbeam’s perspective, an individual’s HL is defined as their ability to comprehend, access, and select health information based on their attitudes and motivations for appropriate self-care and HPBs [36,37]. A prior study supports our findings showing that HL was associated with HPBs and it was the combination of all dimensions of HL—comprehension, accessibility, reading skills, evaluation, and decision-making—and their behaviors could explain 58.0% of the variance in HPBs [17]. Still, many studies have reported that adequate levels of HL were associated with appropriate HPBs [38,39]. Additionally, older adults with adequate HL have been found to promote and encourage their behaviors regarding complex health issues, especially during the COVID-19 pandemic. Although adequate HL has been continuously associated with HPBs during the COVID-19 pandemic in various countries, studies in some contexts have shown the opposite findings, where older adults with adequate HL were not associated with COVID-19 preventive behaviors [20]. The effect of HL on HPBs among older adults with NCDs warrants further examination.

ACPM directly affected HPBs among older adults with NCDs in this study. This finding may be related to the fact that individuals with good accessibility to COVID-19 prevention materials are more likely to have better HPBs, especially during the COVID-19 pandemic. Our findings are consistent with Pechrapa and colleagues [40], who found that older adults in urban areas with good ACPM could access health information, health services, and COVID-19 preventive materials. To prevent and control the spread of COVID-19, the Thai government and healthcare agencies provided some COVID-19 prevention materials, including face masks and soap or alcohol for hand washing to residents of Thailand; however, some people were unable to access free COVID-19 prevention materials. Interestingly, most participants of this study had a monthly income of less than USD 143, which is lower than the average income in Thailand [41]. Thus, it could affect HPBs among older adults who do not have access to preventive materials for COVID-19, particularly older adults in rural communities with lower ACPM. During the new normal of the COVID-19 era, lifestyle and health behaviors changed due to pandemic control measures, significantly affecting older adults with NCDs, particularly people living in poverty [42]. However, health behaviors and disparities in access to healthcare need to be addressed to improve HPBs among all older adults with NCDs [15].

SN also had a positive direct effect on HPBs in our study, in which most of the participants had more social engagement. A previous study supports our findings that older adults who receive good support from family and friends are associated with good COVID-19 preventive behaviors (OR: 2.05, 95% CI: 1.10–3.82) [20]. Maintaining physical and mental well-being throughout life and into old age, SN contributes to HPBs and quality of life [43]. A recent integrative review of factors associated with HPBs during the COVID-19 pandemic revealed that more social engagement with SNs was associated with HPBs among older adults [44]. These findings have been supported with a prior SEM study that examined the factors affecting HPBs among older women in Korea [45]. Social support, including positive social interactions, was an influential factor with a direct effect on HPBs in older women [45]. These findings could explain why social support for older adults is essential for encouraging participation in social activities and maintaining positive relationships and interactions with families, relatives, and peers to establish SNs and improve mental well-being and quality of life [46]. Additionally, SNs are a positive resource that can be obtained through social interactions with family and friendships that enable an individual to live as fully as possible despite any current health conditions [47]. However, many studies revealed that an individual’s lack of SN ties increased their risk of several diseases, morbidity, and mortality [48,49].

The main strength of the current study is its originality, as it was the first study to develop and test a hypothesized causal model of HPBs among older adults with NCDs who lived in Thai urban and rural communities during the new normal post-COVID-19 era. Participants’ ages, education levels, current occupations, and monthly income were positively associated with HPBs. The findings also showed that older adults with NCDs living in urban communities had statistically significantly higher PSE and ACPM scores than older adults in rural communities. Furthermore, HPBs among older adults with NCDs were directly influenced positively by PSE, ACPM, HL, and SN, which could explain 81.0% of the variance in HPBs. These findings highlight significant factors influencing HPBs among urban and rural older adults with NCDs in this transition period of the new normal post-COVID-19 era that health policymakers and healthcare providers can apply to develop proper interventions and healthcare activities according to the local community’s needs and cultural contexts.

There are some limitations to this study. First, the cross-sectional nature limits our ability to establish cause and effect of HPBs among urban and rural older adults with NCDs during the new normal post-COVID-19 era. Second, we collected data using self-reported questionnaires in older adults, and bias in this survey is possible. Third, we did not explore HPBs of older adults in different types of NCDs; future research should compare the different types of older adults with NCDs or focus on specific types of NCDs (e.g., hypertension, diabetes, or heart failure). Finally, the data were collected from only one province in Thailand due to budgetary limitations; thus, caution should be used when generalizing these results to other regions.

## 5. Conclusions

The current study showed that the PSE and ACPM of older adults with NCDs in urban communities were higher than in rural communities. The causal model of HPBs among these populations obtained a good fit with empirical data, which highlighted that PSE, ACPM, HL, and SN directly affect HPBs. Notably, healthcare providers should consider all significant factors to develop comprehensive interventions or healthcare activities for HPBs among older adults with NCDs according to their needs and cultural contexts. Attention should be paid to health behaviors and disparities in both urban and rural communities that affect HPBs among older adults with NCDs in order to encourage HPBs for as long as possible in old age.

## Figures and Tables

**Table 1:** Participants’ demographic data.

Characteristic	Health-Promoting Behaviors			*p*-Value
	Urban (*n* = 125)	Rural (*n* = 125)	Total (*n* = 250)	
	*n* (%)	*n* (%)	*n* (%)	
Sex				
Female	92 (73.60)	67 (53.60)	159 (63.60)	0.446
Age (years): Mean ± SD	70.59 ± 7.44	68.63 ± 7.41	69.61 ± 7.47	0.018
60–69	55 (44.00)	77 (61.60)	132 (52.80)	
70–79	54 (43.20)	34 (27.20)	88 (35.20)	
>80	16 (12.80)	14 (11.20)	30 (12.00)	
Monthly income (US dollars): Mean ± SD	193.58 ± 321.05	70.69 ± 64.91	132.14 ± 239.20	0.026
<143	92 (73.60)	115 (92.00)	207 (82.80)	
144–286	10 (8.00)	10 (8.00)	20 (8.00)	
286–429	6 (4.80)	0 (0.00)	6 (2.40)	
>430	17 (13.60)	0 (0.00)	17 (6.80)	
Type of NCDs (Yes) *				0.176
Heart disease	9 (7.20)	11 (8.80)	20 (8.00)	
Vascular disease	15 (12.00)	8 (6.40)	23 (9.20)	
Diabetes	67 (53.60)	46 (36.80)	113 (45.20)	
Hypertension	82 (65.60)	82 (65.60)	164 (65.60)	
Cancer	1 (0.80)	1 (0.80)	2 (0.80)	
Chronic obstructive pulmonary disease	0 (0.00)	3 (2.40)	3 (1.20)	
Obesity	9 (7.20)	2 (16.0)	11 (4.40)	

NCDs = noncommunicable diseases, SD = standard deviation. * This measure permitted multiple answers.

**Table 2:** Perceived self-efficacy, health literacy, access to COVID-19 preventive material, social networks, and health-promoting behaviors among urban and rural older adults with NCDs.

Variables		Interpretation	Urban (*n* = 125)		Rural (*n* = 125)		Total (*n* = 250)		*p*-Value
			Mean	*SD*	Mean	*SD*	Mean	*SD*	
Perceived self-efficacy (PSE)		High	80.54	17.7	72.42	16.50	76.48	17.55	<0.001
	Nutrition self-efficacy	High	21.09	4.92	19.67	4.17	20.38	4.61	0.014
	Stress management self-efficacy	High	20.12	4.24	17.93	4.28	19.02	4.39	<0.001
	Exercise self-efficacy	Fair	18.55	5.63	15.12	5.87	16.83	5.99	<0.001
	Health practice self-efficacy	High	20.77	4.47	19.69	4.70	20.23	4.61	0.064
Health literacy (HL)		Good	37.50	6.78	37.06	5.94	37.28	6.37	0.586
Access to COVID-19 preventive material (ACPM)		Good	4.46	0.92	3.08	1.30	4.14	1.18	<0.001
Social networks		More social engagement	15.16	5.93	14.99	5.24	15.08	5.59	0.813
Health-promoting behaviors (HPBs)		Good	68.13	7.16	66.73	7.81	67.44	7.51	0.141
	Nutrition	Good	18.91	2.79	18.28	2.97	18.59	2.89	0.084
	Exercise	Fair	6.11	2.12	6.08	1.84	6.09	1.98	0.899
	Smoking	Very Good	9.19	1.77	8.69	1.94	8.94	1.87	0.036
	Alcohol drinking	Very Good	4.61	0.90	4.53	0.98	4.57	0.94	0.505
	Stress management	Fair	5.23	1.04	5.78	1.28	5.50	1.19	<0.001
	Rational drug use	Very Good	9.89	1.85	9.67	2.21	9.78	2.04	0.387
	Preventive COVID-19 infection	Very Good	14.17	1.67	13.68	1.57	13.93	1.64	0.019

**Table 3:** The direct, indirect, and total effects among the variables in the study.

Dependent Variables	*R* ^2^	Effects	Independent Variables			
			ACPM	PSE	SN	HL
HL	0.72	DE	−0.03 * (−0.85)	0.81 *** (13.41)	0.11 * (2.14)	–
		IE	–	–	–	–
		TE	−0.03 * (−0.85)	0.81 *** (13.41)	0.11 * (2.14)	–
HPBs	0.81	DE	0.24 *** (3.55)	0.40 *** (4.32)	0.01 * (0.91)	0.19 ** (2.36)
		IE	−0.01 * (−0.09)	0.15 ** (1.61)	0.02 * (0.39)	–
		TE	0.23 *** (3.46)	0.55 *** (5.92)	0.03 * (0.51)	0.19 ** (2.36)
χ^2^ = 71.936, *df* = 58, *p*-value = 0.103, χ^2^/*df* = 1.240; RMSEA = 0.031; SRMR = 0.042; GFI = 0.964; NFI = 0.964; CFI = 0.993						

ACPM = access to COVID-19 preventive materials, PSE = perceived self-efficacy, SN = social networks, HL = health literacy, HPBs = health-promoting behaviors, DE = direct effect, IE = indirect effect, TE = total effect. * *p* < 0.05, ** *p* < 0.01, *** *p* < 0.001.

## Data Availability

The data sets of this study are not publicly available due to the information that could compromise the research participants’ privacy. However, data may be shared upon request to the authors.
